# Toxicity and Efficacy Evaluation of Soluble Recombinant Ricin Vaccine

**DOI:** 10.3390/vaccines12101116

**Published:** 2024-09-29

**Authors:** Hyeongseok Yun, Hae Eun Joe, Dong Hyun Song, Young-Jo Song, Sunghyun Hong, Chang-Hwan Kim, Na Young Kim, Gyeung Haeng Hur, Chi Ho Yu

**Affiliations:** 1 Defense Advanced Science and Technology Research Institute, Agency for Defense Development, Daejeon 34186, Republic of Korea; 2 ABION Inc., Seoul 08394, Republic of Korea

**Keywords:** plant toxin, ricin, ricin vaccine, R51-3

## Abstract

Background: Ricin, a toxin extracted from the seeds of *Ricinus communis*, is classified as a ribosome-inactivating protein. The A-subunit of ricin shows RNA *N*-glycosidase activity that cleaves ribosomal RNA (rRNA) and exhibits toxicity by inhibiting protein synthesis and inducing vascular leak syndrome. Methods: In this study, we created a truncated version of the previously developed R51 ricin vaccine (RTA 1-194 D75C Y80C) through in silico analysis. Results: The resulting R51-3 vaccine showed a more-than-six-fold increase in soluble protein expression when compared to R51, with over 85% solubility. In a pilot toxicity test, no toxicity was observed in hematological and biochemical parameters in BALB/c mice and New Zealand white rabbits following five repeated administrations of R51-3. Furthermore, R51-3 successfully protected mice and rabbits from a 20 × LD_50_ ricin challenge after three intramuscular injections spaced 2 weeks apart. Similarly, monkeys that received three injections of R51-3 survived a 60 µg/kg ricin challenge. Conclusions: These findings support R51-3 as a promising candidate antigen for ricin vaccine development.

## 1. Introduction

Ricin is a potent toxin, extracted from the seeds of *Ricinus communis*, which is classified as a ribosome-inactivating protein owing to its ability to inhibit protein synthesis [1,2]. The ricin precursor protein is composed of 576 amino acids, which are cleaved post-translation into an enzymatic A subunit (RTA) and a binding B subunit (RTB), linked by a single disulfide bond [3]. RTA (267 amino acids) exhibits RNA *N*-glycosidase activity and cleaves ribosomal RNA (rRNA), thereby leading to protein synthesis inhibition and cell death [4]. RTB (262 amino acids) facilitates binding of ricin to cell surface receptors and its internalization [5]. Ricin intoxication can happen through inhalation, injection, or ingestion, with varying levels of toxicity depending on the exposure route; however, ricin toxicity is highest when inhaled or injected [6]. Symptoms of ricin poisoning generally include inflammation, renal tubular necrosis, hypoglycemia, and gastrointestinal hemorrhages. The estimated lethal dose of ricin for humans is 1–10 µg/kg; because of its high toxicity and potential for mass production, ricin is considered a significant bioterrorism threat [7,8].

Ricin recognizes the sarcin–ricin loop within the GTPase-associated complex of rRNA and hydrolyzes adenine through its *N*-glycosidase activity [9,10]. Previous studies have identified several critical residues in RTA, such as Arg48, Arg134, Arg213, and Arg258, which bind to the rRNA phosphate backbone, and Tyr80, Tyr123, Asn209, and Trp211, which are involved in binding the depurination target adenine [11,12,13,14]. Its catalytic activity significantly depends on Gln177 and Arg180 [15]. Additionally, residues 74–76 of ricin induce vascular leak syndrome (VLS), which is characterized by hypoalbuminemia, peripheral edema, and pulmonary edema [16,17,18].

Previous studies have explored deglycosylated RTA and formaldehyde-inactivated ricin toxoids as vaccine candidates for ricin. However, development was halted due to side effects, such as VLS in animal models, despite these toxoids having reduced toxicity compared to the live toxin [19,20,21]. RiVax—a recombinant vaccine derived from RTA with V76M and Y80A mutations to inactivate the active site—progressed to Phase 1 clinical trials and demonstrated protection against ricin inhalation exposure in rhesus macaques [22]. RVEc—a truncated version of the vaccine with only residues 1–33 and 43–198, after removing the hydrophobic domains of RTA—showed protective effects against ricin toxin [23,24]. Recently, a vaccine integrating the advantages of RiVax and RVEc was developed, with D75A, V76M, and Y80A mutations in RTA 1-198 [25].

Both RVEc and RiVax were evaluated for toxicity in rabbits through intramuscular administration. The maximum vaccine doses used were 200 μg per animal for RVEc and 100 μg per animal for RiVax [26,27]. RVEc and RiVax have successfully completed Phase 1 clinical trials [28,29,30]. RiVax underwent Phase 1B trials and demonstrated safety and the ability to produce ricin-neutralizing antibodies in humans. The next steps for RiVax include pivotal animal efficacy studies and Phase 2 clinical trials to further confirm its safety and effectiveness, correlating immune markers from animal studies with human outcomes. RVEc also completed a Phase 1a trial, showing it to be safe and well tolerated at various doses. Future trials for both vaccines will aim to optimize dosing and further evaluate long-term efficacy to meet biodefense and military needs [31]. 

Despite several ricin vaccines being developed in the United States, there are currently no approved ricin vaccines available for use in South Korea. Therefore, to effectively counter the biological threat posed by ricin, it is necessary to develop and evaluate additional ricin vaccines that can be efficiently produced and supplied. In a previous study, we developed the R51 vaccine by introducing D75C and Y80C mutations in RTA 1-194, creating a disulfide bond between the VLS sequence and the adenine-binding site [32]. In this study, we aimed to further modify R51 by removing the hydrophobic regions at the N-terminus and C-terminus to create R51-3, with enhanced soluble expression. In a preliminary trial for toxicity evaluation, no weight loss was observed in mice after single or five repeated doses of R51-3; in addition, no signs of toxicity were observed in mice or rabbits in this assessment. Furthermore, R51-3-vaccinated mice, rabbits, and monkeys exhibited 100% survival following ricin challenges.

## 2. Materials and Methods

### 2.1. Chemicals, Reagents, Cells, and Animals

The pET-24a vector was purchased from Novagen (Merck, Darmstadt, Germany). Enzymes and *E. coli* T7 Express *lysY*/*I^q^* were obtained from New England Biolabs (Ipswich, MA, USA). All chemicals used for protein purification and antibody titer measurement were purchased from Sigma–Aldrich (St Louis, MO, USA), and all reagents were sourced from Cytiva (Danaher, DC, USA). Gels and buffers for sodium dodecyl-sulfate polyacrylamide gel electrophoresis (SDS-PAGE) were purchased from Thermo Fisher Scientific (Waltham, MA, USA). Bacterial culture media were acquired from Becton and Dickinson (Franklin Lakes, NJ, USA). The 2% aluminum hydroxide (alum) used in animal experiments was obtained from InvivoGen (San Diego, CA, USA). Cell culture materials were purchased from Thermo Fisher Scientific and antibodies were purchased from Abcam (Cambridge, UK). 

All experimental animals were purchased from Orient Bio (Sungnam, Republic of Korea), except for those used for pilot toxicity assessment. The animal experiments were conducted with an equal number of male and female animals (*n* = 2–5 for each), except for the monkeys, where only males were used (*n* = 1–3). All animal tests were approved by the Institutional Animal Care and Use Committee (IACUC).

### 2.2. In Silico Analysis of Protein Structure

The ricin protein sequence (P02879) was obtained from ExPASy (http://www.expasy.org), and the three-dimensional structures (PDB ID: 1IFS, 2AAI) were obtained from the RCSB Protein Data Bank. Discovery Studio 4.5 (BIOVIA, San Diego, CA, USA) was used for protein modeling and analysis of RTA, R51, and truncated R51. Predicted scaled solubility of truncated R51 mutants was measured using Protein-Sol (https://protein-sol.manchester.ac.uk/, accessed on 20 August 2024).

### 2.3. Construction and Soluble Expression of Truncated R51 

To assess the solubility of truncated R51 variants (R51-1 to R51-4), they were generated using R51 as a template for polymerase chain reaction, and products were cloned into pET-28a vector (Novagen, Madison, WI, USA) and pGEX-4T-3 vector (Danaher). For pET-28a, cloning was performed in-frame using *Nde*I and *Xho*I, with the inclusion of a stop codon, to allow for an N-terminal 6×His tag. For pGEX-4T-3, in-frame cloning was done using *Bam*HI and *Not*I, also with the inclusion of a stop codon, to allow for an N-terminal glutathione S-transferase (GST) tag. For toxicity and efficacy evaluation, R51-3 was cloned into pET-24a vector (Novagen) using *Nde*I and *Hin*dIII without any tags, and protein was subsequently purified.

The constructed plasmids were transformed into *E. coli* BL21 (DE3). For protein expression, the bacteria were cultured at 37 °C until an OD600 of 0.4 was reached, induced with 0.5 mM isopropyl thiogalactoside (IPTG), and cultured at 25 °C for an additional 12 h. The cultured *E. coli* were centrifuged at 12,000× *g* at 4 °C for 5 min to collect the cells, which were re-suspended in a lysis buffer (20 mM Tris-Cl pH 8.0, 200 mM NaCl) and lysed using a Qsonica Q700 sonicator (Newtown, CT, USA) with a cycle of 30 s on and 30 s off for a total of 5 min on ice, at 40% amplitude. The soluble lysate and pellet were separated by centrifugation at 12,000× *g* at 4 °C for 15 min. Soluble protein expression was analyzed by loading the lysate onto a NuPAGE^TM^ 4–12% Bis-Tris gel and running the gel in NuPAGE^TM^ MES SDS running buffer. The gel was stained with Coomassie blue to observe protein expression.

Soluble expressions of R51 and R51-3, as well as inclusion bodies, were compared based on a previous study [33]. A 1 L culture induced with 0.5 mM IPTG was centrifuged at 6000× *g* at 4 °C for 20 min to collect the supernatant. The lysate was prepared by resuspending the cell pellet in lysis buffer (50 mM TRIS-Cl pH 8.5, 100 mM NaCl, 1 mM phenylmethylsulfonyl fluoride, and 5 mM ethylenediaminetetraacetic acid [EDTA]) and sonicated with a cycle of 30 s on and 30 s off for a total of 5 min on ice, at 40% amplitude, followed by centrifugation at 12,000× *g* at 4 °C for 15 min. The pellet was washed twice with distilled water, and the sample was solubilized in solubilization buffer (50 mM TRIS-Cl [pH 8.5], 30% trifluoroethanol, 3 M urea, and 1 mM dithiothreitol) at 23–25 °C for 2 h. The supernatant and inclusion bodies were separated by centrifugation at 15,000× *g* at 4 °C for 20 min and analyzed using 12% SDS–PAGE. The band intensity of each fraction was measured using ImageJ (US National Institute of Health, Bethesda, MD, USA) [34]. For the Western blot analysis, samples were separated by NuPAGE^TM^ 4–12% Bis-Tris gel and transferred to a PVDF membrane. The membrane was then blocked with 5% skim milk in PBST (PBS with 0.05% Tween-20) at 23–25 °C for 30 min, incubated with HRP Anti-6×His tag antibody (Abcam) at 23–25 °C for 1 h with gentle rocking, followed by chemiluminescence detection.

### 2.4. Protein Purification

Ricin was purified following established protocols [35]. Approximately 25 g of decorticated castor bean was blended with 250 mL of 0.15M NaCl, 0.01M phosphate-buffered saline (PBS) (pH 7.4) buffer for 30 min. The mixture was stirred at 4 °C for 16 h, and the supernatant was collected after centrifugation at 10,000× *g* at 4 °C for 30 min. The supernatant was adjusted with 60% *w*/*v* ammonium sulfate, followed by centrifugation at 15,000× *g* at 4 °C for 30 min to remove the supernatant. The pellet was dissolved in 25 mL of 0.01M PBS buffer (pH 6.8), and then dialyzed three times against 4 L of 0.01 M PBS (pH 6.8). Then, the solution was applied to a hydroxyapatite column using an AKTA avant^TM^ system (Danaher) and eluted with a linear gradient of 0.01–0.3 M PBS pH 6.8. The ricin-containing elute was concentrated using a Centricon (Merck, Darmstadt, Germany), followed by gel filtration. The column was loaded with the sample and eluted with 0.02 M PBS buffer (pH 7.4), with fractions collected at one-column intervals.

Purification of R51-3 for efficacy and toxicity assessment was conducted by ABION (Seoul, Republic of Korea). The pET-24a R51-3 plasmid was transformed into *E. coli* T7 Express *lysY*/*I^q^* (NEB), and the cells were grown in Select APS Super Broth (BD) with 0.5% glycerol and induced with 1 mM IPTG at 25 °C for 5 h. Cells were harvested, re-suspended in 20 mM Tris-Cl pH 7.0 and 1 mM EDTA buffer, and lysed using a microfluidizer at 18,000 psi for three cycles to harvest the lysate. The lysate was centrifuged at 15,000× *g* at 4 °C for 20 min, and the supernatant was harvested. To remove impurities, the supernatant was acidified to pH 3.5 using acetic acid, then filtered through a 0.45 µm filter after centrifugation at 15,000× *g* at 4 °C for 20 min. Then, the supernatant was loaded onto an SP Sepharose Fast Flow (FF) column and eluted with 50 mM sodium acetate pH 5.8. The elute was adjusted to 1 M ammonium sulfate by adding 3 M ammonium sulfate and adjusting the pH to 7.0 with NaOH. Subsequently, the solution was loaded onto a Phenyl Sepharose FF column and eluted with 50 mM sodium phosphate buffer (pH 7.0). Finally, the solution was loaded onto a Q Sepharose FF column, and the unbound flow-through was collected.

### 2.5. Toxicity Assessment

To observe weight changes after single and repeated administrations in mice, 20 µg R51-3 and 10 µL of 2% alum (InvivoGen) were suspended in 100 µL PBS. Both the control and vaccine groups consisted of 10 8-week-old ICR mice (five males and five females). The vaccine was administered intramuscularly (IM) with 50 µL injected into each thigh, and repeated injections were given weekly for 5 weeks to monitor weight changes. To observe the effects of repeated vaccine administration on body weight changes in BALB/c mice (five males and five females) and New Zealand white rabbits (five males and five females), vaccine solution of 200 µg of R51-3 and 100 µL of 2% alum in 1 mL PBS was prepared. In BALB/c mice, 50 µL vaccine was injected into each thigh (total 100 µL, 20 µg R51-3 and 10 µL of 2% alum), while 500 µL vaccine (100 µg R51-3 and 50 µL of 2% alum) was injected IM into the right hind leg of rabbits. The control group was injected with PBS, while the adjuvant group received alum mixed in PBS without R51-3. Pilot toxicity assessments were conducted by DT&CRO (Yongin, Republic of Korea) using BALB/c mice (five males and five females) and New Zealand white rabbits (five males and five females). The adjuvant solution was prepared by suspending 100 µL of 2% alum in 1 mL PBS; the vaccine solution was prepared by suspending 200 µg of R51-3 and 100 µL of 2% alum in 1 mL PBS. For the BALB/c mice, 100 µL of PBS, adjuvant (10 µL of 2% alum), or vaccine (20 µg R51-3 and 10 µL of 2% alum) solutions were administered IM, with 50 µL injected into each thigh. For New Zealand white rabbits, 500 µL of PBS, adjuvant (50 µL of 2% alum), or vaccine (100 µg R51-3 and 50 µL of 2% alum) solutions were administered IM into the right hind leg. After five weekly injections, blood was collected the following day. To obtain serum, a portion of the collected blood was allowed to clot at 23–25 °C for 1 h, followed by centrifugation at 2000× *g* at 4 °C for 15 min. The supernatant was then used for analysis. Biochemical analysis was performed using the Hitachi 7180 Chemistry Analyzer (Hitachi, Tokyo, Japan), and hematological analysis was performed using the XN-1000 Hematology Analyzer (Sysmax, Kobe, Japan). All vaccine toxicity tests were approved by the IACUC of DT&CRO (approval number: mouse, 23N0139; rabbit, 23N0137). 

For the histopathological analysis, the liver was fixed for more than 24 h in 10% formalin at a volume 30–50 times greater than the tissue volume following necropsy. Subsequently, the left lateral lobe of the liver and the left/right medial lobes including the gallbladder were sectioned into 3–4 mm slices. These slices were placed into cassettes and subjected to secondary fixation in 10% formalin depending on the fixation state. The sectioned tissues were paraffin-embedded using a tissue processor (VIP6, Sakura Finetek Japan, Tokyo, Japan) and an embedding center (TEC5, Sakura Finetek Japan), and sections of 3–5 µm thickness were prepared using a microtome (RM2245, Leica, Wetzlar, Germany). The sections were stained and mounted using an automatic stainer and coverslipper (Prisma™ Glas™ g2, Sakura Finetek Japan).

### 2.6. Efficacy Assessment

The vaccine solution was prepared by suspending 20 µg R51-3 and 10 µL of 2% alum in 100 µL PBS. For the efficacy evaluation, ten 8-week-old ICR mice (five males, five females) were immunized with three IM injections of 100 µL vaccine (20 µg R51-3 and 10 µL of 2% alum) at 2-week intervals, with 50 µL administered into each thigh. The control group consisted of four ICR mice (two males, two females) that received PBS IM. To determine the median lethal dose (LD_50_) of ricin, survival of 14-week-old ICR mice (*n* = 50) was observed after IM injection of ricin, and the data were analyzed using the Probit analysis software (EPA Probit Analysis Program, version 1.5). At 2 weeks after the last vaccine administration, 50 µL blood was collected via orbital puncture and survival was monitored for 7 days post-ricin challenge. For the LD_50_ determination in rabbits, the survival of 3–4-kg New Zealand white rabbits (*n* = 16) was observed following an IM injection of ricin. New Zealand white rabbits (five males, five females) were first immunized with three IM injections of 500 µL vaccine (100 µg R51-3 and 50 µL of 2% alum) at 2-week intervals, administered into the right hind leg. The control group consisted of four New Zealand white rabbits (two males, two females) that received PBS IM. At 2 weeks after the last vaccine administration, 200 µL blood was collected from the ear vein of rabbits, and survival was monitored post-ricin challenge. Three male rhesus monkeys (4–10 kg) were IM administered with 500 µL vaccine (100 µg R51-3 and 50 µL of 2% alum) at 2-week intervals, administered into the right hind leg. A male rhesus monkey was IM administrated with 500 µL PBS. At 2 weeks after the last vaccine administration, 200 µL of blood was collected via venipuncture, and survival was monitored post-ricin challenge. The efficacy assessment was conducted according to the Agency for Defense Development IACUC approved protocols: ADD-IACUC-22-13 (mouse), ADD-IACUC-22-14 (rabbit), and ADD-IACUC-23-03 (monkey). 

### 2.7. Antibody Titer and TNA

The measurement of anti-ricin antibody titer from serum was done according to established protocols [36,37]. Ricin was diluted to a concentration of 2 μg/mL in 0.05 M carbonate–bicarbonate buffer (pH 9.6), and 50 μL was dispensed into each well of a 96-well plate. Then, the plate was incubated at 4 °C for 16 h. After removing the buffer, the wells were washed three times with 300 mL PBST using an AquaMax microplate washer (Molecular Devices, San Jose, CA, USA), and blocked with blocking buffer (PBST with 5% skim milk) at 37 °C for 1 h. The blocking buffer was used based on previous literature [37,38]. After three washes with 300 μL PBST, the serum from mice was diluted to 1/50 in blocking buffer, serially diluted two-fold in the blocked wells, and incubated at 37 °C for 1 h. The wells were washed three times with PBST, followed by incubation with anti-mouse IgG horseradish peroxidase (HRP) diluted 1:10,000 in blocking buffer at 37 °C for 1 h. Then, the plate was washed three times with PBST. For detection, 100 μL of substrate solution (0.05 M phosphate–citrate buffer pH 5.0, 0.4 mg/mL O-phenylenediamine dihydrochloride, and 0.003% hydrogen peroxide) was added to each well and incubated in the dark at 23–25 °C for 30 min. The reaction was stopped by adding 50 μL of 3 M sulfuric acid to each well, and absorbance was measured at 492 nm. The same method was used to measure ricin antibody titers in rabbits and monkeys, using anti-rabbit IgG HRP and anti-monkey IgG HRP, respectively. 

The toxin neutralizing assay (TNA) was conducted based on the existing literature to measure the cell viability rates after ricin treatment [39,40]. Jurkat cells (ATCC TIB-152) were cultured in RPMI with 10% fetal bovine serum and 1% penicillin/streptomycin at 37 °C with 5% CO_2_. For TNA, 2 × 10^4^ cells per well were seeded in a 96-well plate and incubated overnight. 

The following day, a mixture of 0.5 ng/mL ricin and 1% serum was prepared in an Eppendorf tube and incubated at 37 °C for 1 h. Subsequently, this mixture was added to Jurkat cells. After 24 h of incubation, EZ-Cytox (DoGenBio, Seoul, Republic of Korea) was added according to the manufacturer’s instructions. Then, the cells were incubated for an additional 2 h at 37 °C with 5% CO_2_, after which absorbance was measured at 450 nm.

### 2.8. Statistical Analysis

All data are presented as means ± standard errors of the mean. Statistical significance was determined using a two-tailed Student *t*-test and one-way analysis of variance (ANOVA) with post hoc Fisher tests. Statistical significance was defined as *p* < 0.05.

## 3. Results

### 3.1. Structure and Expression of the Truncated R51

**Figure 1:**
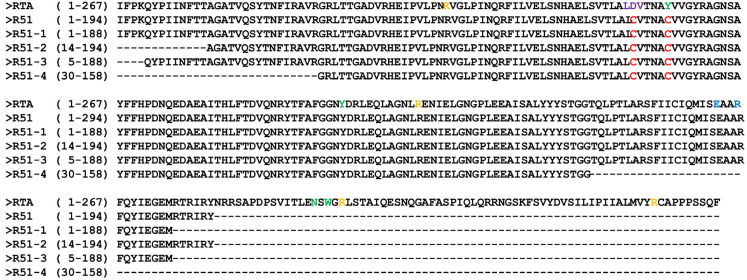
Amino acid sequences of RTA, R51, and truncated R51 mutants. Mutation site (red), rRNA binding site (yellow), VLS site (purple), adenine binding site (green), and catalytic activity site (blue) are shown. Abbreviation: VLS, vascular leak syndrome.

**Figure 2:**
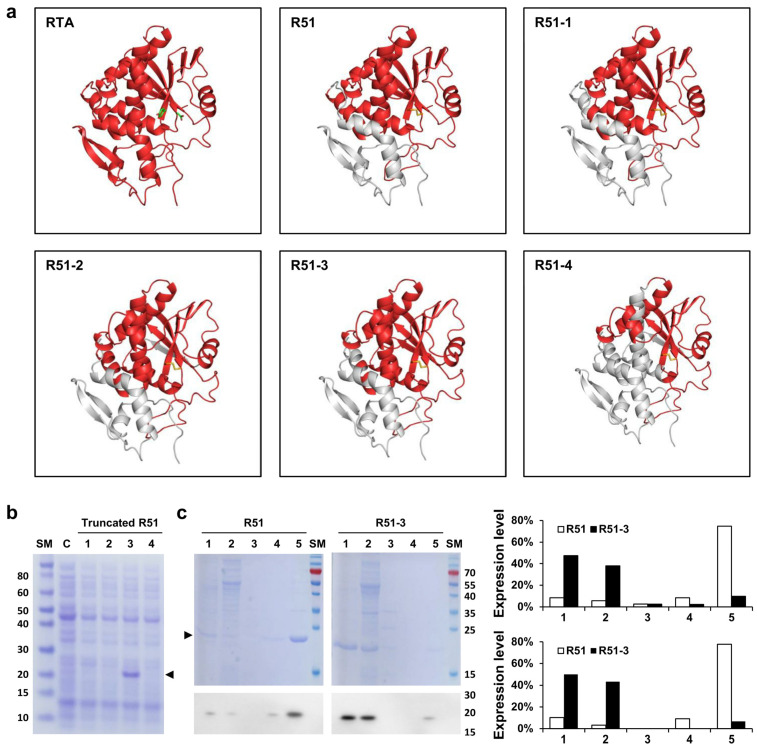
In silico analysis and solubility expression of truncated R51 mutants. (**a**) Predicted structures of the truncated R51 mutants using in silico tools. The D75 and Y80 of RTA are indicated in green, and the disulfide bond between D75C and Y80C is shown in yellow. The gray region indicates the removed portions. (**b**) Soluble expression of truncated R51 observed in Coomassie staining. Arrows indicate truncated R51 level. Lanes: 1, cell lysate of the R51-1 expression strain; 2, cell lysate of the R51-2 expression strain; 3, cell lysate of the R51-3 expression strain; 4, cell lysate of the R51-4 expression strain. (**c**) Comparison of soluble expression of R51 and R51-3. The figure shows Coomassie staining results in the top panel and Western blot results using the 6×His antibody in the bottom panel. Lanes: 1, media after protein induction; 2, supernatant after 1st lysis; 3, supernatant after washing with distilled water; 4, supernatant after 2-h reaction in solubilization buffer; 5, inclusion bodies. The expression levels are presented as percentages of the total protein expression, measured based on the Coomassie staining results (top) and Western results (bottom). Abbreviations: C, cell alone; RTA, ricin subunit A; SM, size marker.

The R51 protein, which is an RTA 1-194 D75C Y80C mutant, contains hydrophobic regions at both the N-terminus and C-terminus (Figure 1). To increase the solubility of R51, a truncated version was created by removing these hydrophobic regions. Predicted scaled solubility measured using Protein-sol was 0.380 for R51, and 0.417, 0.423, 0.476, and 0.510 for the four truncated candidates R51-1 to R51-4, respectively (Table 1) [41]. Three-dimensional structural modeling predicted that R51-1, R51-2, and R51-3 maintained relatively stable structures (Figure 2a). When the truncated R51 variants tagged with 6×His were induced at 25 °C, the soluble expression of R51-3 was observed, while R51-1 showed weak soluble expression (Figure 2b). However, soluble expression was not detected for R51-2 and R51-4. To improve solubility, the truncated R51 variants were expressed as N-terminal GST fusions, resulting in an increase in the soluble expression of R51-1, with levels comparable to those of R51-3 (Figure S1a). However, R51-2 and R51-4 still did not exhibit any detectable soluble expression. In the Coomassie staining results, we observed that 85% of R51-3 was soluble, whereas only 14% of R51 was in soluble form. 

Since the soluble expression of R51-3 was higher at 25 °C than at 37 °C, we expressed untagged R51-3 at 25 °C and then performed purification for the pilot toxicity test (Figure S1b). After purification, the yield of R51-3 was 14.85 mg/L, showing a higher purification yield compared to R51, which had a yield of 1.5 mg/L (Figure S1c).

### 3.2. Weight Changes in Mice after Single and Repeated Doses of R51-3

**Figure 3:**
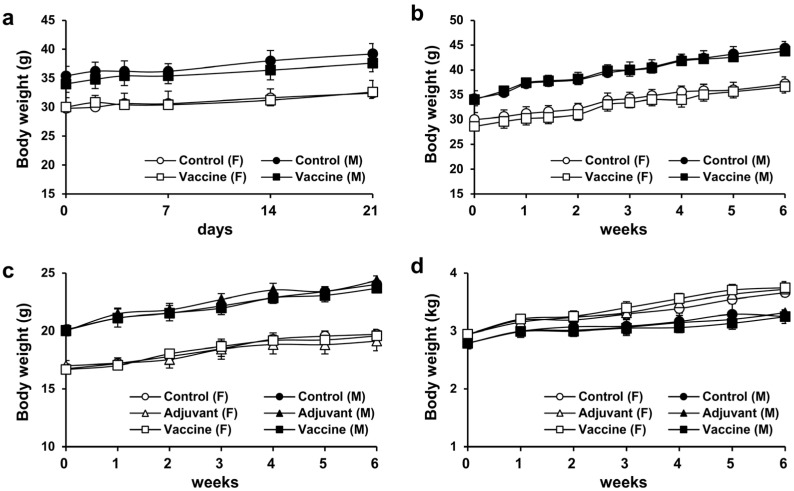
Body weight changes after single and repeated administrations of R51-3 vaccine. (**a**) Body weight changes in ICR mice after a single administration. Approximately 20 µg of R51-3 and 10 µL of 2% alum were suspended in 100 µL of PBS and administered intramuscular route. (**b**) Body weight changes in ICR mice after five weekly administrations. No significant differences in body weight were observed between the control and vaccine groups, regardless of sex. (**c**) Body weight changes in BALB/c mice after five weekly administrations. No significant differences in body weight were observed between the control, adjuvant, and vaccine groups. (**d**) Body weight changes in New Zealand white rabbits after five weekly administrations. The control group received five intramuscular injections of 500 µL PBS at one-week intervals, the adjuvant group (*n* = 5) received 50 µL alum in 500 µL PBS, and the vaccine group (*n* = 5) received 100 µg R51-3 and 50 µL 2% alum in 500 µL PBS. No significant differences in body weight were observed between the control, adjuvant, and vaccine groups. All data are presented as means ± standard errors of the mean. Statistical analysis between two groups by gender was performed using a two-tailed Student *t*-test (**a**,**b**), while analysis among the three groups was conducted using one-way analysis of variance (**c**,**d**). Abbreviations: alum, aluminum hydroxide; F, female; M, male; PBS, phosphate-buffered saline; control, PBS-treated group (*n* = 5); adjuvant, adjuvant-treated group (*n* = 5); vaccine, vaccine-treated group (*n* = 5).

To assess the toxicity of R51-3 in ICR mice, weight changes over a 3-week period were monitored after a single-dose administration of 20 µg of R51-3 with alum as an adjuvant. No significant weight differences were observed between the control group and the vaccine-treated group, or between female and male mice (Figure 3a). Similarly, no weight differences were observed after five weekly administrations of R51-3 at the same dosage in ICR mice (Figure 3b). Additionally, body weight changes were monitored in BALB/c mice and New Zealand white rabbits following five administrations at one-week intervals. In BALB/c mice, groups were divided into control (PBS), adjuvant (10 µL 2% alum), and vaccine (20 µg R51 and 10 µL 2% alum) groups, and no differences in body weight were observed between the administration groups in both female and male mice (Figure 3c). Likewise, in New Zealand white rabbits, no significant differences in body weight were observed between the vaccine group (100 µg R51 and 50 µL 2% alum), the adjuvant group (50 µL 2% alum), or the control group, in both female and male rabbits (Figure 3d).

### 3.3. Pilot Toxicity Assessment

**Figure 4:**
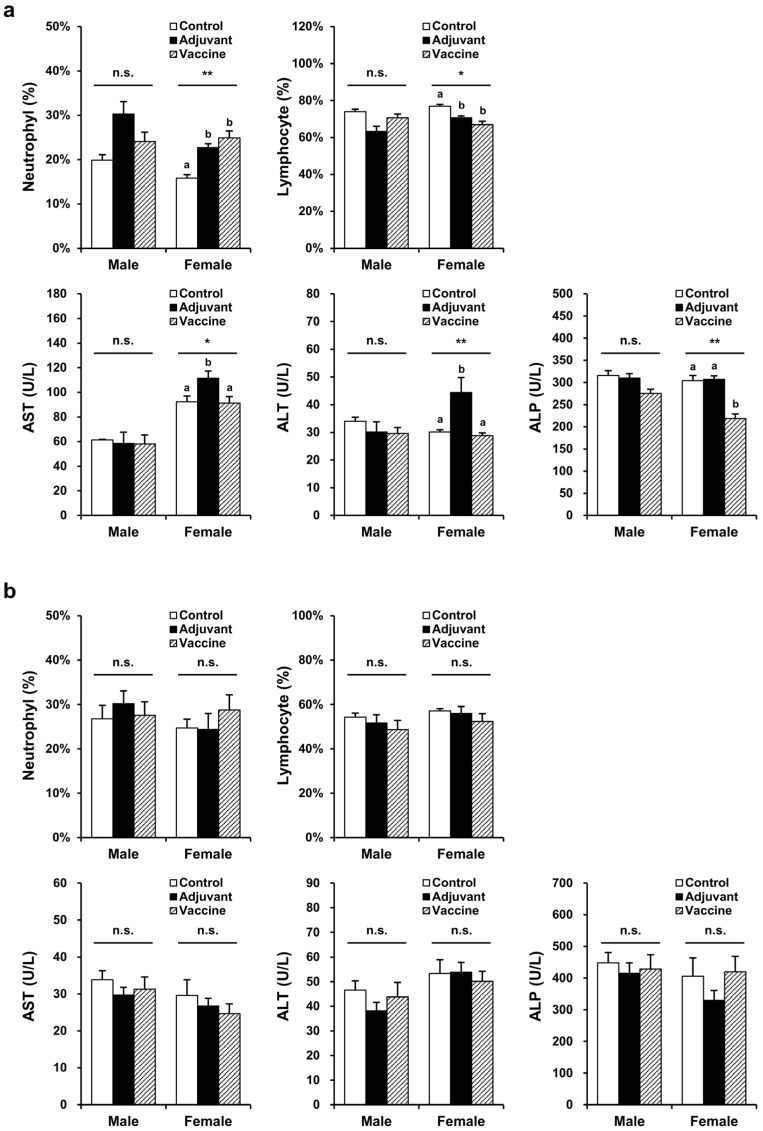
Hematological and biochemical parameters showing significant differences in the pilot toxicity test. (**a**) Changes observed in BALB/c mice after five vaccine administrations. The control group (*n* = 5) received five intramuscular injections of 100 µL PBS at one-week intervals, the adjuvant group (*n* = 5) received 10 µL alum in 100 µL PBS, and the vaccine group (*n* = 5) received 20 µg R51-3 and 10 µL 2% alum in 100 µL PBS. * *p* < 0.05; ** *p* < 0.01; n.s., not significant. (**b**) Changes observed in New Zealand white rabbits after five vaccine administrations. The control group (*n* = 5) received five intramuscular injections of 500 µL PBS at one-week intervals, the adjuvant group (*n* = 5) received 50 µL alum in 500 µL PBS, and the vaccine group (*n* = 5) received 100 µg R51-3 and 50 µL 2% alum in 500 µL PBS. All data are presented as means ± standard errors of the mean. Statistical analysis was performed by one-way analysis of variance, followed by post hoc Fisher test. ^a,b^ Groups showing statistically significant differences (*p* < 0.05) in the post hoc Fisher test. Abbreviations: ALP, alkaline phosphatase; alum, aluminum hydroxide; ALT, alanine aminotransferase; AST, aspartate aminotransferase; PBS, phosphate-buffered saline.

Toxicity was evaluated in BALB/c mice and New Zealand white rabbits, with each species divided into three groups: control (five males, five female), adjuvant (five males, five female), and vaccine (five males, five female). The control (PBS), adjuvant (10 µL of 2% alum), and vaccine (20 µg R51-3 and 10 µL of 2% alum) groups were each administered IM at a volume of 100 µL to BALB/c mice with 50 µL injected into each thigh. For New Zealand white rabbits, control (PBS), adjuvant (50 µL of 2% alum), and vaccine (100 µg R51-3 and 50 µL of 2% alum) groups were each administered 500 µL of solution IM into the right hind leg. In male mice, no changes were observed in the hematological parameters, including the red blood cell (RBC) count, hemoglobin (HGB), hematocrit level (HCT), mean corpuscular volume (MCV), mean corpuscular hemoglobin level (MCH), and mean cell hemoglobin concentration (MCHC), as well as in the platelet (PLT) count, reticulocytes (RET), white blood cell (WBC) count, neutrophils (NEU), lymphocytes (LYM), monocytes (MONO), eosinophils (EOS), and basophils (BASO) (Table S1). However, in female mice, the NEU and LYM levels showed significant changes, with NEU increasing to 24.9 ± 1.6% in the vaccine group compared to 15.8 ± 0.8% in the control group (*p* < 0.05), and LYM decreasing to 66.9 ± 1.8% in the vaccine group compared to 76.9 ± 1.0% in the control group (*p* < 0.05) (Figure 4a). In rabbits, no significant differences were observed in all hematological parameters, including the prothrombin time (PT) and the activated partial thromboplastin time (APTT). In male mice, no significant differences were observed in blood biochemical parameters, including the aspartate aminotransferase (AST), alanine aminotransferase (ALT), alkaline phosphatase (ALP), total bilirubin (T-BIL), total cholesterol (T-CHO), triglyceride (TG), glucose (GLU), blood urea nitrogen (BUN), creatinine (CREA), total protein (TP), albumin (ALB), inorganic phosphorus (IP), calcium (Ca), sodium (Na), potassium (K), and chloride (Cl) levels (Table S2). As shown in Figure 4, in female mice, AST and ALT levels were significantly higher in the adjuvant group compared to those in the control group (AST: 111.5 ± 5.9 U/L vs. 92.4 ± 4.6 U/L, *p* < 0.05; ALT: 44.4 ± 5.4 U/L vs. 30.2 ± 0.7 U/L, *p* < 0.05). However, in the vaccine group, the AST and ALT levels (91.4 ± 5.1 U/L and 28.8 ± 1.0 U/L, respectively) showed similar levels to those in the control group (92.4 ± 4.6 U/L and 30.2 ± 0.7 U/L, respectively). Additionally, the ALP levels significantly differed, showing a decrease in the vaccine group (219 ± 10 U/L) compared to those in the control group (304 ± 12 U/L) (*p* < 0.05). In rabbits, no significant differences were observed in any blood biochemical parameters, including the albumin/globulin (A/G) ratio, γ-glutamyltranspeptidase (GGT) levels, and globulin (GLO) levels. In BALB/c mice and New Zealand white rabbits, no differences were observed in body weight or organ-to body weight ratios between the control, adjuvant, and vaccine groups in both male and female subjects (Figure S2).

### 3.4. Ricin Defense Capability, Antibody Titer, and Generation of Neutralizing Antibodies after Ricin Immunization in Mice

**Figure 5:**
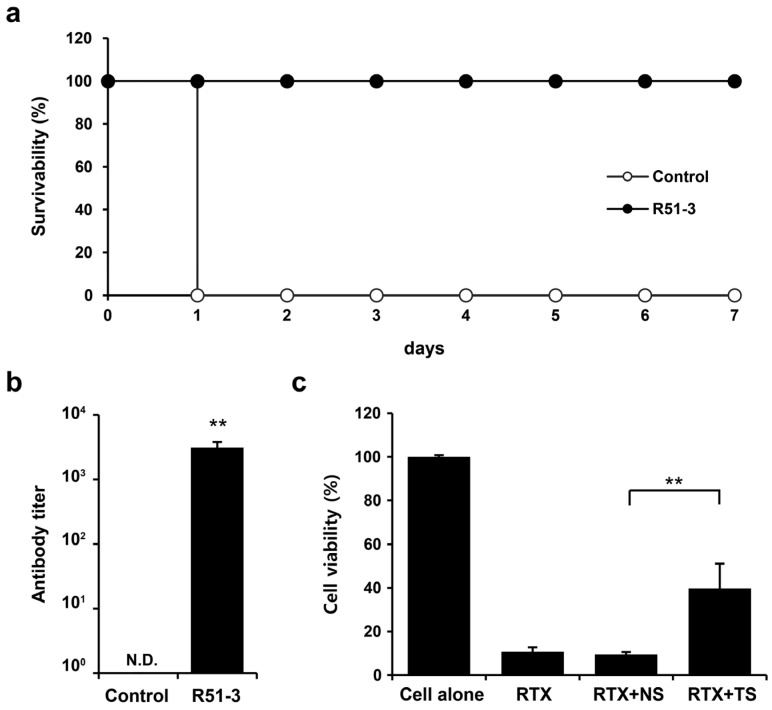
Efficacy of R51-3 vaccine in ICR mouse. (**a**) Survival rates after 20 × LD_50_ ricin challenge in the control and vaccine groups. The control group received three PBS injections at 2-week intervals (two males, two females), whereas the R51-3 group received three vaccine injections at 2-week intervals (five males, five females). (**b**) Ricin antibody titers in the control and vaccine groups. Blood was collected via orbital puncture at 2 weeks after the last vaccine injection, and serum was isolated for antibody titer measurement. ** *p* < 0.01. (**c**) Ricin toxin neutralizing assay results using the serum. All data are presented as means ± standard errors of the mean. Statistical analysis was performed by two-tailed Student *t*-test. Abbreviations: N.D., not detected; PBS, phosphate-buffered saline; RTX, ricin toxin-treated cells; RTX + NS, ricin toxin and normal (PBS-treated mice) serum-treated cells; RTX + TS, ricin toxin and test (R51-3 vaccinated mice) serum-treated cells.

Ricin was purified for use in the challenge tests, based on the existing literature [35]. Considering the vaccine administration period, ricin was IM administered to 14-week-old ICR mice, and the observed LD_50_ was 0.572 µg/animal (Figure S3a). After administering 100 µL vaccine (20 µg of R51-3 and 10 µL of 2% alum) to 8-week-old ICR mice three times at 2-week intervals, and subsequently challenging them with 20 × LD_50_ ricin, all control group mice died within 1 day, while the vaccine group exhibited a 100% survival rate (Figure 5a). Serum collected via orbital puncture before the challenge was analyzed by ELISA, which revealed an antibody titer of 3085 ± 703 (Figure 5b). A TNA was conducted to determine neutral antibody generation in vaccinated mice. The cell viability rate did not differ between the group treated with ricin and 1% mouse normal serum (RTX + NS; 9.4 ± 1.2%) and the ricin-only treated group (RTX; 10.7 ± 2.7%) (Figure 5c). In contrast, the cell viability rate in the group treated with 1% serum from the vaccinated mice (RTX + TS) significantly increased to 39.7 ± 11.4% (*p* < 0.01).

### 3.5. Vaccine Efficacy in Rabbits and Monkeys

**Figure 6:**
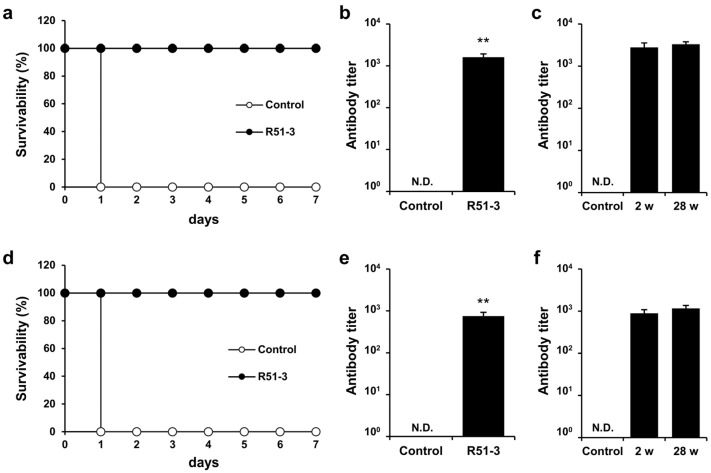
Efficacy of R51-3 vaccine in New Zealand white rabbits and Rhesus monkeys. (**a**) Survival rates after 20 × LD_50_ ricin challenge in rabbits. The control group received three PBS injections at 2-week intervals (two males, two females), whereas the R51-3 group received three vaccine injections at 2-week intervals (five males, five females). (**b**) Ricin antibody titers in the control and vaccine groups of rabbits. Blood was collected from the ear vein of rabbits at 2 weeks after the last vaccine injection. ** *p* < 0.01. (**c**) Long-term antibody titers analysis in rabbits challenged with ricin. Two female rabbits were sampled for blood prior to vaccination (control), and after receiving three doses of the vaccine at 2-week intervals, blood samples were taken two weeks after the final dose (2 w), immediately followed by a 20 × LD_50_ ricin challenge. Six months after the ricin challenge, blood samples were collected again (28 w) to analyze ricin antibody titers. (**d**) Survival rates after 60 µg/kg ricin challenge in monkeys. The control group received three PBS injections at 2-week intervals (one male), while the R51-3 group received three vaccine injections at 2-week intervals (three males). (**e**) Ricin antibody titers in control and vaccine groups in monkeys. Blood was collected from the venipuncture at 2 weeks after the last vaccine injection. (**f**) Long-term antibody titer analysis in monkeys challenged with ricin. Two male monkeys were sampled for blood prior to vaccination (control), and after receiving three doses of the vaccine at 2-week intervals, blood samples were taken two weeks after the final dose (2 w), immediately followed by a 60 µg/kg ricin challenge. Six months after the ricin challenge, blood samples were collected again (28 w) to analyze ricin antibody titers. All data are presented as means ± standard errors of the mean. Statistical analysis was performed by two-tailed Student *t*-test. Abbreviation: N.D., not detected; PBS, phosphate-buffered saline.

In New Zealand white rabbits, the LD_50_ for ricin was 2.11 µg/kg when administered IM (Figure S3b). After immunizing rabbits with 500 µL vaccine (100 µg of R51-3 and 50 µL of 2% alum) three times at 2-week intervals and challenging them with 20 × LD_50_ ricin, all rabbits in the control group died on day 1, whereas all vaccinated rabbits survived (100% survival rate) (Figure 6a). Serum collected via the ear vein from these rabbits showed an antibody titer of 1611 ± 313 in ELISA (Figure 6b). Rhesus macaques were used as non-human primates for the trial. After immunization with the same dose, the antibody titer in the vaccinated group was measured at 752 ± 174. All vaccinated monkeys survived a 60 µg/kg ricin challenge, in contrast to all control monkeys that died within 1 day (Figure 6d,e). To access long-term immunity, ricin antibody titers were analyzed 28 weeks after the final vaccine dose in both rabbits and monkeys, despite being challenged with ricin two weeks after the last vaccination. As shown in Figure 6c,f, the ricin antibody titers were sustained even after 28 weeks.

## 4. Discussion

We predicted that removing the hydrophobic amino acids from the N-terminus and C-terminus of R51 could increase its solubility. Differing from the predicted scaled solubility results, soluble expression of R51-2 and R51-4 was not observed. Analysis of the inclusion bodies from His-tagged truncated R51 which was conducted in the BL21-CodonPlus (DE3)-RIPL strain that provides additional tRNAs for rare codons, revealed that R51-2 was barely expressed and not properly expressed even in this strain (Figure S1d). Unlike R51-2, R51-4 was expressed normally but failed to form a soluble protein. When an N-terminal GST fusion was used to increase soluble expression, the soluble expression of R51-1 increased to a level similar to that of R51-3, but R51-4 remained insoluble, suggesting that the protein could not maintain its proper structure (Figure S1a).

The structure and neutralizing epitopes of ricin have been extensively investigated. The C-terminal 199–267 residues of RTA can reduce thermal stability and do not contain many neutralizing epitopes [42,43]. After administration of anti-CD25 RTA-based immunotoxin, B-cell epitopes were identified at residues 161–175 in patients with Hodgkin’s lymphoma [44]. In addition, T-cell epitopes were identified at residues 174–184 [45] when ricin was used as the immunotoxin. The R51-3 developed in this study retains residues 161–184 involved in antibody formation, but four residues were removed: Asn209 and Trp211, which are involved in adenine binding; and Arg213 and Arg258, which bind to the rRNA phosphate backbone. Despite retaining the catalytic active site, R51-3 exhibited no observed toxicity due to the removal or alteration of residues inducing VLS and binding sites for phosphate and adenine. Although R51-3 lacks residues 187–198—another critical epitope for RTA that neutralizes antibody formation—efficacy tests in ICR mice showed that the antibody titer reached 1791 ± 878, even with 0.5 µg R51-3, with an 80% survival rate following a 20 × LD_50_ ricin challenge (Figure S4).

When developing R51, we observed changes in vaccine efficacy depending on the adjuvant used, and confirmed that alum was the most effective adjuvant for R51 [32]. To shorten the vaccination period, two doses of 0.5 µg R51-3 were administered at 2-week intervals, and the consequent antibody titer significantly changed to 303 ± 194 (*p* < 0.01), with a reduced survival rate of 20%. These results align with existing findings that the optimal condition for recombinant ricin vaccine administration via the IM route involves three doses using alum as an adjuvant [22,23,24].

Ricin toxin A (RTA) exerts its toxic effects by using its RNA N-glycosidase activity to cleave a specific adenine residue from the 28S rRNA, thereby inactivating the ribosome and halting protein synthesis, ultimately leading to cell death [4]. To attenuate the toxicity of R51, we previously introduced a Y80C mutation at the adenine binding site. When the toxicity was tested in Jurkat cells, RTA exhibited cytotoxicity at concentrations above 0.37 μg/mL, whereas R51 did not show any cytotoxic effects even at a concentration of 10 μg/mL [32]. Furthermore, using a human in vitro translation kit, we observed that while RTA inhibited green fluorescent protein expression, R51 showed no such inhibitory effects. Therefore, we predicted that truncated forms of R51, including R51-3, would not exhibit cytotoxicity and did not perform additional tests.

Pilot toxicity assessments show that an increase in neutrophils and a decrease in lymphocytes were observed in female mice in both the adjuvant and vaccine groups compared to the control group (Figure 4). Some reports have indicated an increase in neutrophils and a decrease in lymphocytes after alum administration; however, these changes may be attributed to immune or inflammatory responses at the injection site [46,47]. Granulomatous inflammation and inflammatory infiltration observed at the injection sites in both the adjuvant and vaccine groups were likely attributed to the immune response triggered by the adjuvant rather than vaccine-induced toxicity, as there was no difference in body weight between groups in female mice (Figure S2a). Although changes in neutrophils and lymphocytes in male mice were not statistically significant across the control, adjuvant, and vaccine groups, an increase in neutrophils and a decrease in lymphocytes were observed in the adjuvant group compared to the control. This difference appears to be due to the alum administration.

The AST and ALT levels are indicators of liver disease, with ALT being a particularly reliable indicator for hepatocyte injuries. In our toxicity assessment, female mice showed elevated AST and ALT levels after adjuvant administration compared to those in the control group, while the vaccine group showed no such significant differences, indicating that the liver injury was not caused by the vaccine. ALP is generally found in the liver and bones. Although the ALP levels decreased significantly in vaccinated female mice, the absence of changes in organ-to-body weight ratio and histopathological lesions, except for slight infiltrations of immune cells in the liver, suggests that ALP variations were not indicative of toxicity [48]. In both mice and rabbits, the ALP levels were elevated, likely due to the measurement using the Japan Society of Clinical Chemistry (JSCC) method. Previous studies have reported that ALP levels measured by the JSCC method are approximately three times higher than those measured by the International Federation of Clinical Chemistry and Laboratory Medicine method [49]. The reference range for ALP in BALB/c mice was 331 ± 67 (*n* = 39) for males and 346 ± 67 (*n* = 40) for females. In New Zealand white rabbits, the ALP levels were 352 ± 136 (*n* = 48) for males and 399 ± 218 (*n* = 55) for females.

The ricin LD_50_ for rhesus monkeys is 5–15 µg/kg via aerosol exposure and 9 µg/kg via intraperitoneal injection [50,51,52]. Due to the increased demand for non-human primates following the emergence of SARS-CoV-2 and restrictions on wildlife trade in major exporting countries, the non-human primate supply has decreased substantially, limiting our study to the available rhesus monkeys [53]. Based on the efficacy results of R51-3 in mice, we anticipated that R51-3 would exhibit efficacy comparable to RiVax in rhesus monkeys, and accordingly, we set the ricin dose to ensure 100% lethality in the control group [54]. Our results indicated that a dose of 60 µg/kg of ricin administered IM to rhesus monkeys led to the death of one monkey in the control group within 20 h, while all three vaccinated monkeys survived.

## 5. Conclusions

In this study, we developed a soluble recombinant ricin vaccine R51-3, which did not exhibit toxicity. By removing only 10 amino acids from R51, we increased the soluble fraction of recombinant ricin from 14% to 85%. The R51-3 ricin vaccine, with alum as an adjuvant, showed no observed toxicity in mice and rabbits, and demonstrated protective efficacy against ricin in mice, rabbits, and rhesus monkeys. We believe that R51-3 has the potential to be developed into a ricin vaccine, following current Good Manufacturing Practices and Investigational New Drug application procedures.

## Figures and Tables

**Table 1:** Solubility prediction of truncated R51 mutants using Protein-sol.

Protein	Predicted Scaled Solubility	pI
RTA	0.367	6.040
R51	0.380	6.380
R51-1	0.417	5.410
R51-2	0.423	6.010
R51-3	0.476	5.190
R51-4	0.510	4.890

pI, isoelectric point.

## Data Availability

All data are available in the main text or the Supplementary Materials.

## Supplementary Materials

The following supporting information can be downloaded at: https://www.mdpi.com/article/10.3390/vaccines12101116/s1. Figure S1. Soluble expression of truncated R51 under different conditions, and purification results. (**a**) Changes in soluble expression of truncated R51 as a result of the modification of the N-terminal tag. The cells induced at 25 °C were disrupted by sonication, and after centrifugation, only the soluble lysate was analyzed. Lanes 1 to 4 correspond to R51-1 to R51-4, respectively. Arrows indicate the expressed proteins. (**b**) Soluble expression of R51-3 at different induction temperature. The induced cells were disrupted by sonication and then centrifuged to separate the soluble lysate and pellet. Lanes: 1, 6 h incubation at 25 °C; 2, 3 h incubation at 30 °C; 3, 1.5 h incubation at 37 °C. (**c**) Purification results of R51, R51-3, and ricin. Lanes: 1, R51; 2, R51-3; 3, ricin. (**d**) Analysis of inclusion bodies from truncated R51. The BL21-CodonPlus (DE3)-RIPL cells induced at 25 °C were sonicated and centrifugated to obtain the pellet. The pellet was washed with wash buffer (20 mM Tris-Cl pH 8.0, 200 mM NaCl, 0.5% Triton X-100), followed by centrifugation to isolate the inclusion bodies. Lanes 1 to 4 correspond to R51-1 to R51-4, respectively. Abbreviations: C, cell alone; GST, glutathione S-transferase; 6×His, 6×Histidine; SM, size marker. Figure S2. Body weight changes following repeated administration of the R51-3 vaccine in the toxicity assessment. (**a**) Body weight changes in BALB/c mice after five weekly administrations. There were no significant body weight changes among the control, adjuvant, and vaccine groups during the administration for toxicity assessment. (**b**) Body weight changes in New Zealand white rabbits after five weekly administrations. There were no significant body weight changes among the control, adjuvant, and vaccine groups during the administration for toxicity assessment. Abbreviations: F, female; M, male; control, phosphate-buffered saline-treated group (*n* = 5); adjuvant, adjuvant-treated group (*n* = 5); vaccine, vaccine-treated group (*n* = 5). Figure S3. Determination of ricin LD_50_. (**a**) Determination of ricin LD_50_ in ICR mice. Ricin was diluted in 100 µL of PBS and administered via the intramuscular route. Survival was observed for 2 weeks. (**b**) Determination of ricin LD_50_ in NZW rabbits. Ricin was diluted in 500 µL of PBS and administered IM. Survival was observed for 2 weeks. Abbreviations: IM, intramuscularly; NZW rabbit, New Zealand white rabbit; PBS, phosphate-buffered saline. Figure S4. Survival rates and antibody titers in ICR mice according to the number of R51-3 vaccine doses administered. (**a**) Survival rates after 20 × LD_50_ ricin challenge according to the number of vaccine doses. The control group received three PBS injections at 2-week intervals, and the vaccine group received 0.5 µg of R51-3 and 10 µL of 2% alum suspended in 100 µL of PBS. All groups consisted of five female ICR mice. The ricin challenge was performed two weeks after the last injection. (**b**) Ricin antibody titers according to the number of vaccine doses. Blood was collected via orbital puncture 2 weeks after the last vaccine injection, and serum was isolated for antibody titer measurement. *** p* < 0.01. All data are presented as means ± standard errors of the mean. Statistical analysis was performed by two-tailed Student *t*-test. Abbreviations: LD_50_, median lethal dose; PBS, phosphate-buffered saline. Table S1. Hematological parameters in the control, adjuvant, and vaccine groups. Table S2. Serum biochemical parameters in the control, adjuvant, and vaccine groups.
