# Supplementary figures and images for: Toxicity and Efficacy Evaluation of Soluble Recombinant Ricin Vaccine
Source: Vaccines (Basel). 2024 Sep 29;12(10):1116. doi: 10.3390/vaccines12101116 (PMC11511097; doi:10.3390/vaccines12101116)

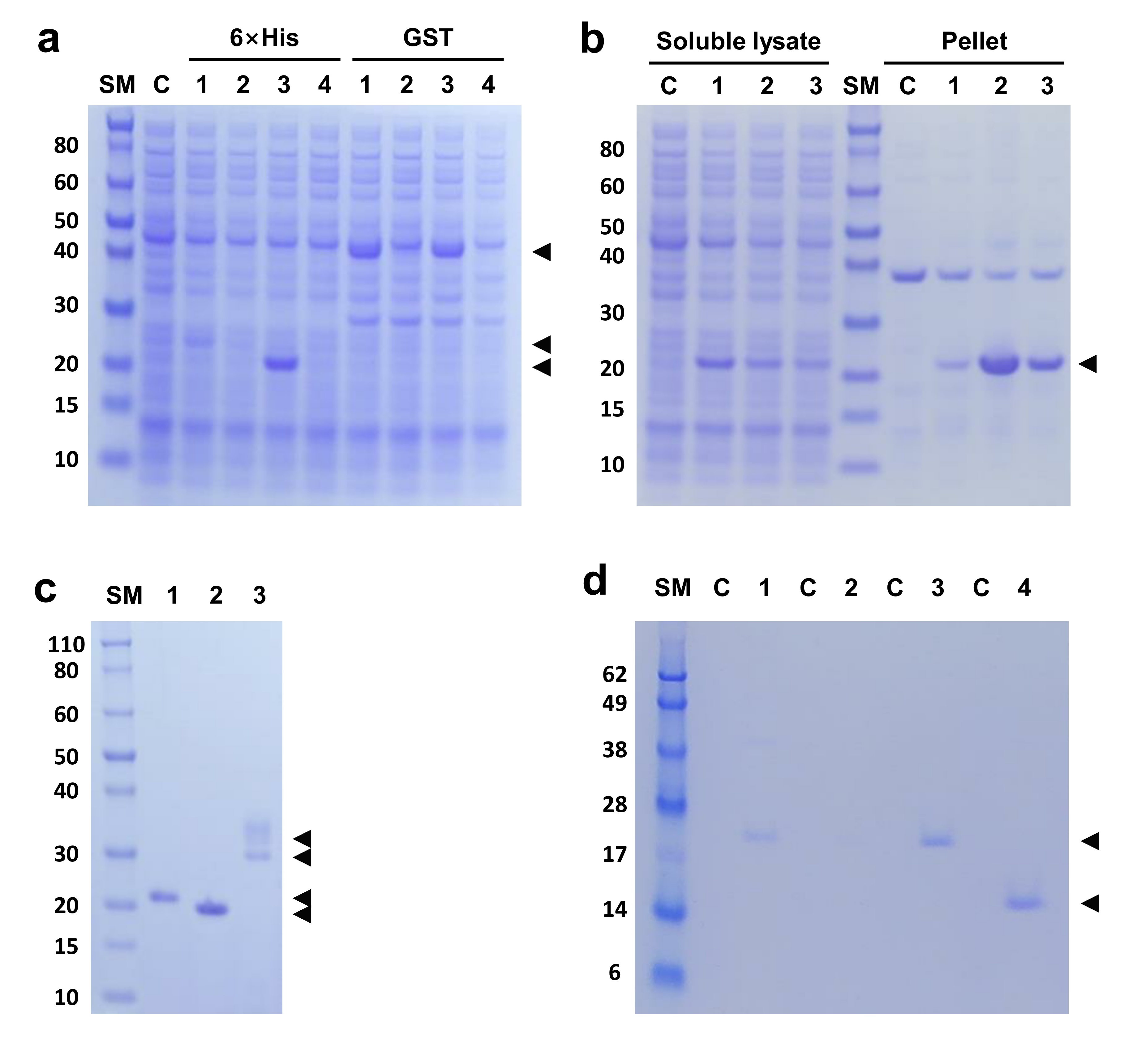

Supplement: Supplementary file 1 [file vaccines-12-01116-s001.zip › Figure S1.tif]

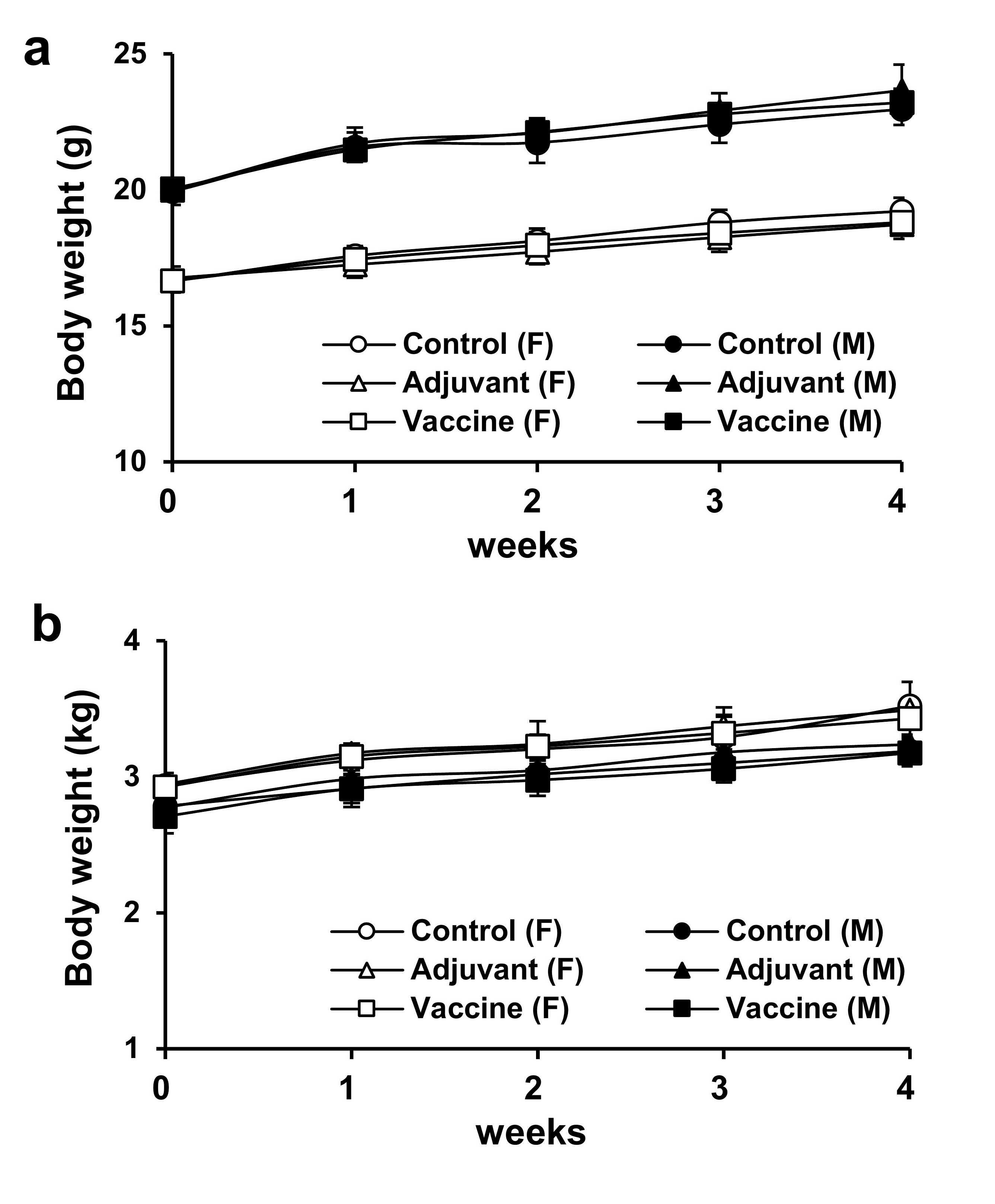

Supplement: Supplementary file 1 [file vaccines-12-01116-s001.zip › Figure S2.tif]

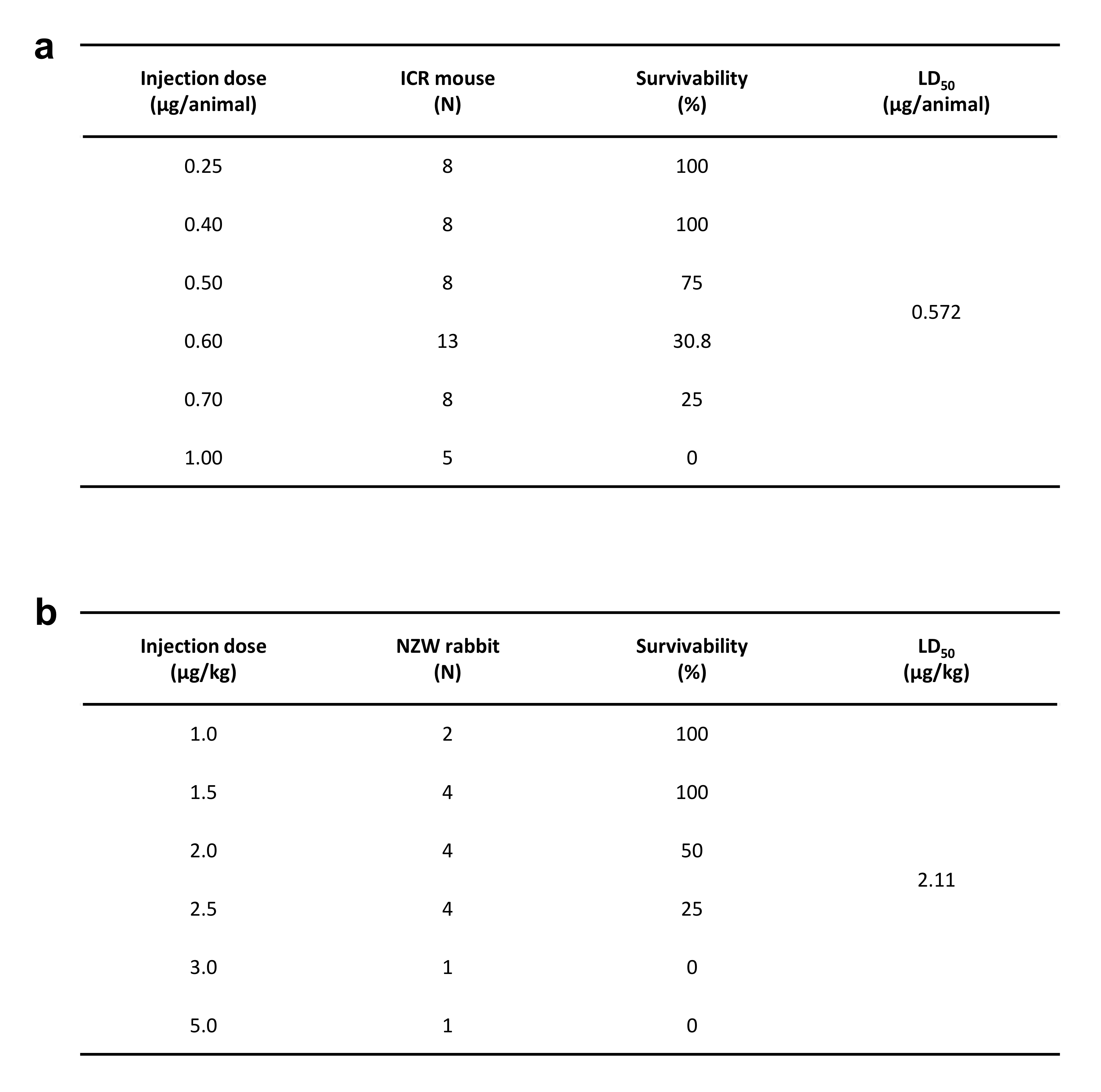

Supplement: Supplementary file 1 [file vaccines-12-01116-s001.zip › Figure S3.tif]

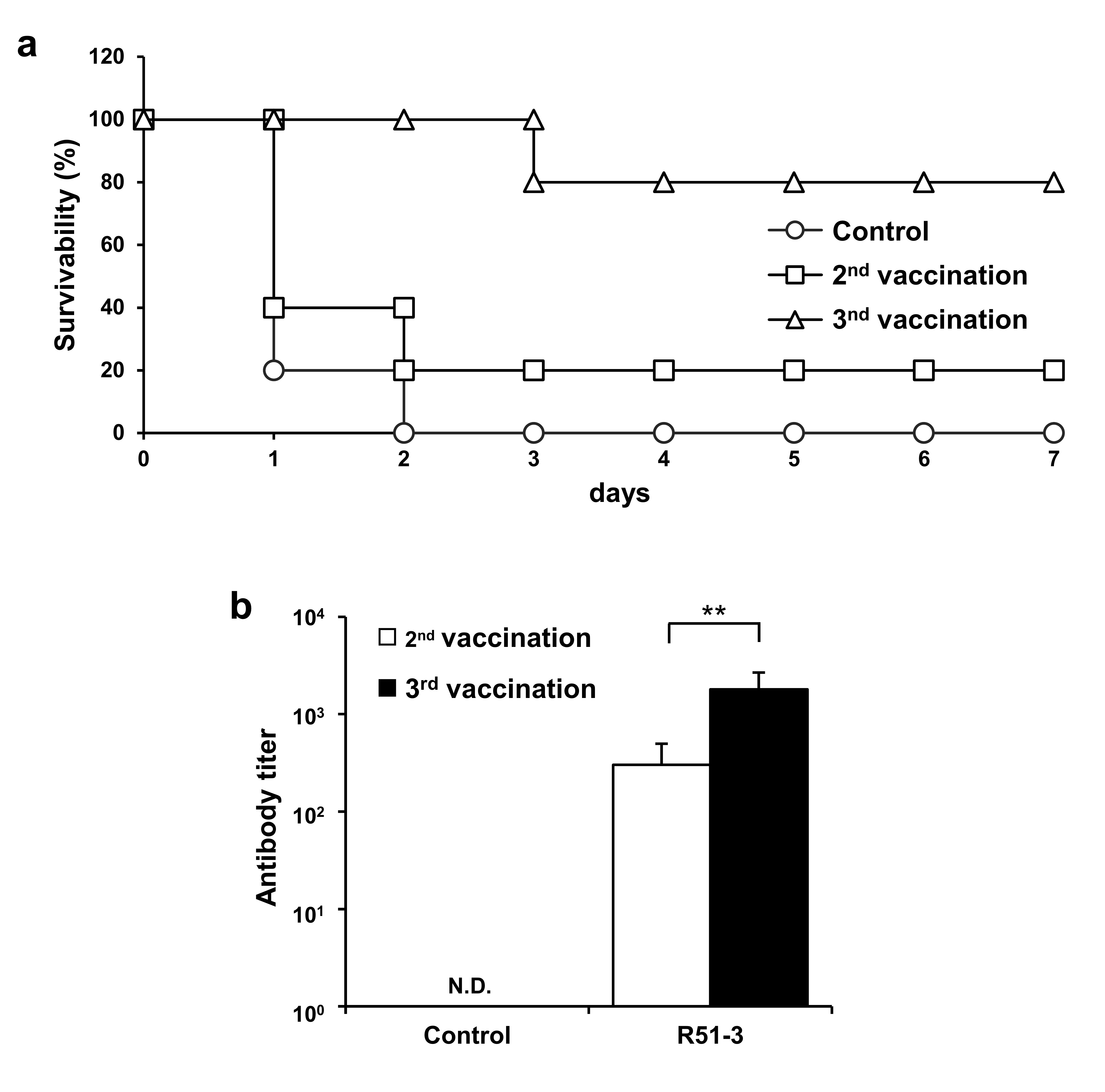

Supplement: Supplementary file 1 [file vaccines-12-01116-s001.zip › Figure S4.tif]

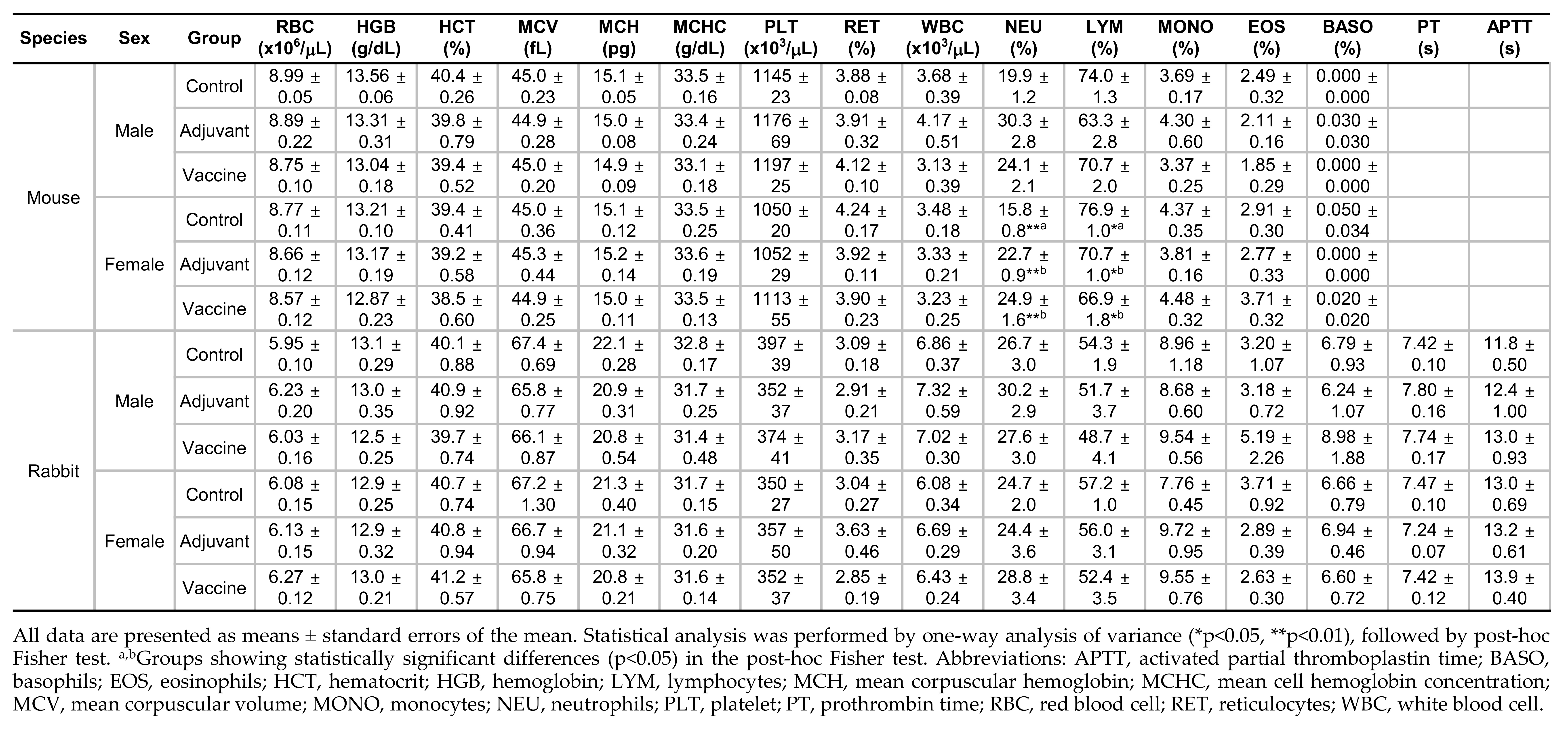

Supplement: Supplementary file 1 [file vaccines-12-01116-s001.zip › Table S1.tif]

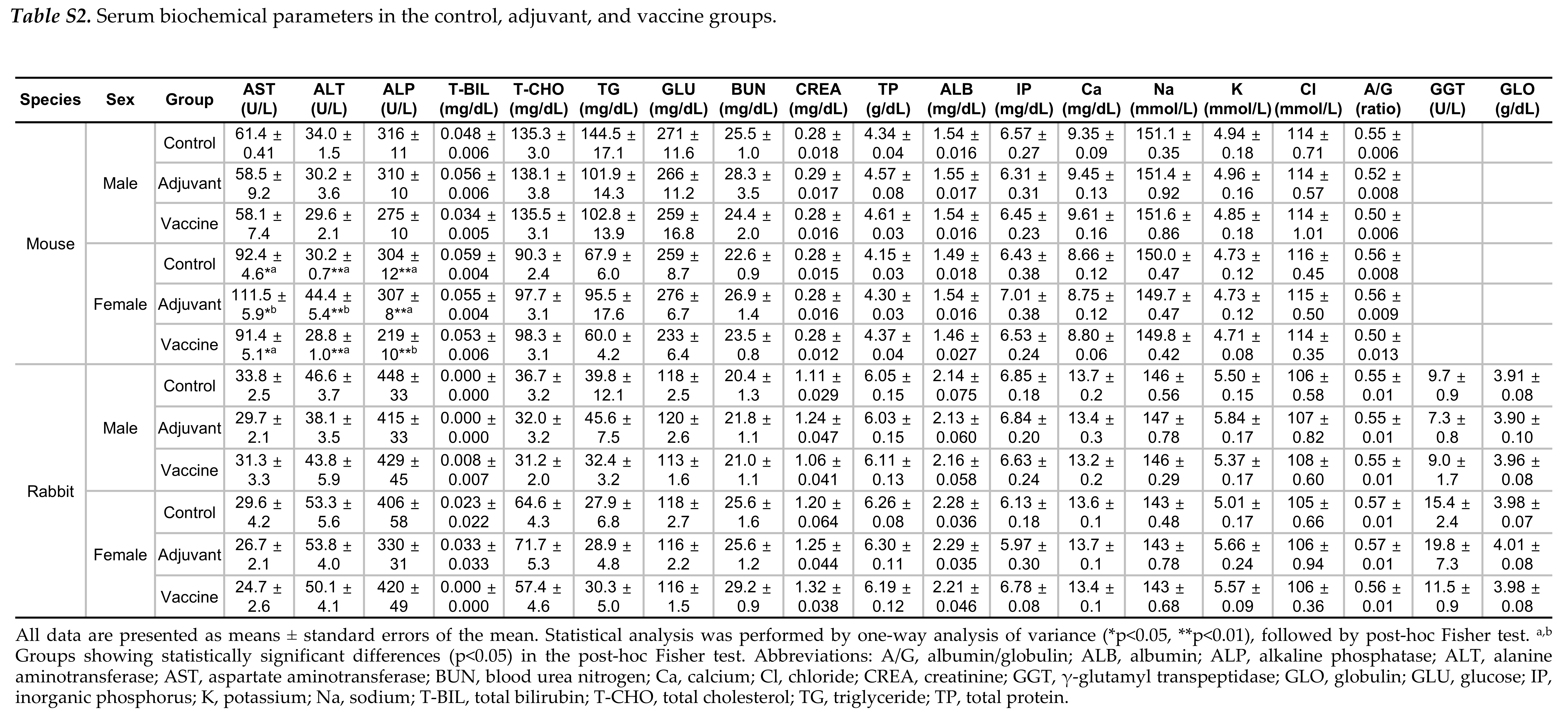

Supplement: Supplementary file 1 [file vaccines-12-01116-s001.zip › Table S2_revised.tif]
